# The Role of Multifidus in the Biomechanics of Lumbar Spine: A Musculoskeletal Modeling Study

**DOI:** 10.3390/bioengineering10010067

**Published:** 2023-01-04

**Authors:** Kuan Wang, Zhen Deng, Xinpeng Chen, Jiang Shao, Lulu Qiu, Chenghua Jiang, Wenxin Niu

**Affiliations:** 1 Shanghai YangZhi Rehabilitation Hospital (Shanghai Sunshine Rehabilitation Center), School of Medicine, Tongji University, Shanghai 200092, China; 2 Shanghai Baoshan Hospital of Integrated Traditional Chinese and Western Medicine, Shanghai 201900, China; 3 Laboratory of Rehabilitation Engineering and Biomechanics, Department of Rehabilitation Sciences, School of Medicine, Tongji University, Shanghai 200092, China

**Keywords:** musculoskeletal modeling, multifidus, lumbar spine, joint loading, biomechanics

## Abstract

Background: The role of multifidus in the biomechanics of lumbar spine remained unclear. Purpose: This study aimed to investigate the role of multifidus in the modeling of lumbar spine and the influence of asymmetric multifidus atrophy on the biomechanics of lumbar spine. Methods: This study considered five different multifidus conditions in the trunk musculoskeletal models: group 1 (with entire multifidus), group 2 (without multifidus), group 3 (multifidus with half of maximum isometric force), group 4 (asymmetric multifidus atrophy on L5/S1 level), and group 5 (asymmetric multifidus atrophy on L4/L5 level). In order to test how different multifidus situations would affect the lumbar spine, four trunk flexional angles (0°, 30°, 60°, and 90°) were simulated. The calculation of muscle activation and muscle force was done using static optimization function in OpenSim. Then, joint reaction forces of L5/S1 and L4/L5 levels were calculated and compared among the groups. Results: The models without multifidus had the highest normalized compressive forces on the L4/L5 level in trunk flexion tasks. In extreme cases produced by group 2 models, the normalized compressive forces on L4/L5 level were 444% (30° flexion), 568% (60° flexion), and 576% (90° flexion) of upper body weight, which were 1.82 times, 1.63 times, and 1.13 times as large as the values computed by the corresponding models in group 1. In 90° flexion, the success rate of simulation in group 2 was 49.6%, followed by group 3 (84.4%), group 4 (89.6%), group 5 (92.8%), and group 1 (92.8%). Conclusions: The results demonstrate that incorporating multifidus in the musculoskeletal model is important for increasing the success rate of simulation and decreasing the incidence of overestimation of compressive load on the lumbar spine. Asymmetric multifidus atrophy has negligible effect on the lower lumbar spine in the trunk flexion posture. The results highlighted the fine-tuning ability of multifidus in equilibrating the loads on the lower back and the necessity of incorporating multifidus in trunk musculoskeletal modeling.

## 1. Introduction

Low back pain is a common musculoskeletal problem and is the leading cause of disability worldwide [1]. Spinal degeneration is one of the main reasons of low back pain with or without radiculopathy. As an important structure of the low back, lumbar spine plays a role in supporting the upper body weight (UBW). Therefore, it is crucial to understand the biomechanics of lumbar spine for prevention of low back pain.

A mechanical factor takes part in the process of spinal degeneration. The load on lumbar spine may vary on the basis of the position of the trunk. To maintain the equilibrium state of the lumbar spine, many muscles across several spinal levels keep active to generate proper force to balance the loads produced by the upper body mass. One of the paraspinal muscles, multifidus, is located close to the dorsal side of the spine. Along with semispinalis and rotators, the multifidus is regarded as the transversospinales, which fills up the groove between spinous process and transverse process [2].

The principal fascicles of the lumbar multifidus attach the lateral side of the spinous process to the mamillary processes, iliac crests, and dorsal surface of sacrum [3]. The fascicles of lumbar multifidus span 2 to 5 spinal levels and have a high cross-sectional area, allowing them to produce large forces. Due to this morphology, the lumbar multifidus is involved in stabilizing the lumbar spine, and dysfunction of the lumbar multifidus is considered as one important factor leading to low back pain [4]. Therefore, lots of therapeutic exercises and electrical simulation therapies were designed to restore the function of multifidus [5,6,7]. To further understand the role of multifidus in lumbar stabilization, a number of clinical studies were carried out to assess the function of lumbar multifidus through ultrasound, computed tomography, magnetic resonance imaging, and electromyography [8,9]. Although these studies provide us with much valuable information on the anatomy and morphology of the lumbar multifidus, the mechanical role of the lumbar multifidus in various human postures still needs to be identified.

Musculoskeletal modeling based on rigid body dynamics is a useful method to investigate human biomechanics, and it is widely applied in the field of medicine [10], ergonomics [11], exoskeleton development [12], and training machine designment [13]. More particular, the musculoskeletal model is popular in the fields of orthopedics and sports due to its simplicity in mechanical data collecting and estimation [14,15]. A lot of modeling systems were designed for musculoskeletal modeling and simulation, e.g., Opensim [16], Anybody [17], MASS [18], and a number of approaches were proposed for individualized modeling [19,20]. In practice, subject-specific musculoskeletal modeling is always performed to reflect subject’s mechanical condition [21]. Then, the model takes kinematics as input and can be used to estimate muscle activity and joint loads, which helps to analyze the effect of different surgical approaches [22], rehabilitation exercises [23], and injury prevention methods [24]. 

For diverse research objects, many researchers have developed a variety of trunk musculoskeletal models with varying levels of modeling details. For example, Christophy et al. [25] constructed a detailed musculoskeletal model for the lumbar spine. A musculoskeletal model of the fully articulated thoracolumbar spine and rib cage was developed by Bruno et al. [26]. Bae et al. [27] analyzed the biomechanics of lumbar spine during golf swing movement using a musculoskeletal model with simplified trunk muscles. Jo and Chae [28] investigated the load during occupational activities using a simplified musculoskeletal model in conjunction with a finite model. In the abovementioned studies, some authors only took the major muscle groups of the spine into consideration but neglected small muscles like the multifidus [25,26], whereas other authors constructed trunk models with detailed description of all musculature [27,28]. These differences in modeling increased the heterogeneity of the results and complicated the comparison between models. Therefore, it is necessary to investigate the role of multifidus in the modeling of lumbar spine to improve the comparability of trunk models.

Asymmetric muscle atrophy is another question involving the multifidus [29]. Hyun et al. [30] found asymmetry of the multifidus muscles in patients who had unilateral lumbosacral radiculopathy with herniated disc. Battié et al. [31] reported that the ratio of functional cross-sectional area to total cross-sectional area of multifidus at the level and below the level of herniation was found to be smaller on the side of the herniation than that on the unaffected side. Although these researchers confirmed the existence of multifidus asymmetric atrophy in low back pain patients with or without radiculopathy, the biomechanical influence of this phenomenon remained unclear. On the other hand, inconsistent results were reported about multifidus asymmetric atrophy in low back pain patients. For example, Rezazadeh et al. [32] found no correlation between the multifidus muscle’s cross-sectional area or thickness variations among the L4-L5 and L5-S1 levels and disability index score in chronic low back pain patients. As one of the muscles closest to the lumbar vertebra, the multifidus is expected to have a crucial impact on the biomechanics of the lower back. Thus, to explain the relationship between the reduction in cross-sectional area of the multifidus and clinical manifestations of low back pain with or without radiculopathy, the effect of asymmetric multifidus atrophy on the biomechanics of the lumbar spine needs to be identified.

Therefore, the aim of this study was twofold. The first objective was to examine the role of multifidus in the modeling of lumbar spine. The second objective was to investigate the influence of asymmetric multifidus atrophy on the biomechanics of lumbar spine. To achieve these goals, a set of open-sourced trunk musculoskeletal models were modified to simulate the various conditions of multifidus. This study was expected to provide insights in the detailed modeling of lumbar spine and to highlight the role of multifidus in the biomechanics of lower back.

## 2. Methods

In this study, five conditions of multifidus were considered in the trunk models according to the research objectives. All of the models were built based on a set of open-sourced trunk musculoskeletal models [33], which included 250 trunk musculoskeletal models representing 250 individuals. These models were constructed based on the subject-specific morphological information of bone and muscles extracted from computed tomography scans. The models were the extension of the thoracolumbar spine musculoskeletal model built by Bruno et al. [26], which was well validated against in vivo measures of intradiscal pressure (IDP), vertebral loading, and myoelectric activity. The lumbar multifidus in the original intact model includes 25 fascicles located at each side of the spinal process, originating from the mammillary processes of the lumbar vertebrae, and posterior surface of sacrum and iliac crest, and inserting onto the spinous process of lumbar vertebrae 2–5 levels above. The intact model also modeled thoracic multifidus with one fascicle at each level and each side of the thoracic spine. The fascicles of thoracic multifidus span one spinal levels which originate from the transverse processes and insert onto the spinous processes of the vertebrae above. Each fascicle has its own maximum isometric force derived from the cross-sectional area computed from tomography scans [26]. Since the models were parameterized, the maximum isometric force of each fascicle in these models can be modified. The detailed modification process was described below. 

The first set of models were the control group which included the full modeling of multifidus at all spinal levels. The second group simulated the condition that did not consider the effect of multifidus at all spinal levels, thus the maximum isometric force of all multifidus muscle in this group was set to 0. These two groups of models were used to examine whether or not the incorporation of multifidus in the trunk musculoskeletal models had an effect on the biomechanics of lumbar spine. To further explain the modeling effect of multifidus, a third group was also added, in which the maximum isometric forces of all multifidus muscle were set to half of their original values.

The fourth and fifth set of models simulated the conditions of asymmetric multifidus atrophy because the multifidus atrophy is related to the low back pain or disc herniation. Unilateral multifidus atrophy was modeled on the L4/L5 or L5/S1 level, which have the highest incidence rate of disc degeneration [34]. Thus, the fourth groups simulated the unilateral multifidus atrophy on the L4/L5 level, and the fifth groups simulated the unilateral multifidus atrophy on the L5/S1 level. To simulate unilateral multifidus atrophy, the muscle bundles of multifidus of the right side across the L4/L5 or L5/S1 were selected, and the maximum isometric forces of these muscle bundles were set to half of their original values.

**Figure 1:**
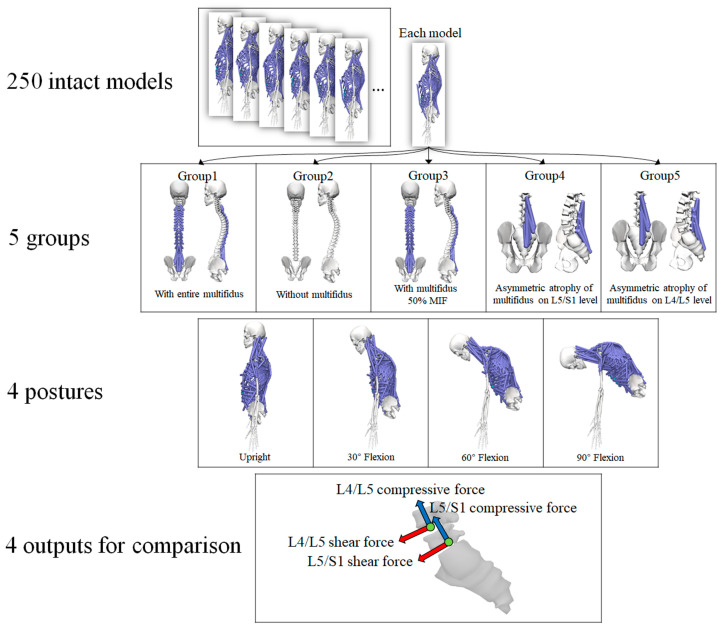
The scenarios for simulation (MIF, maximum isometric force).

The multifidus is the extensor muscle of spine [3]. Hence, trunk flexion postures with four flexional angles (0°, 30°, 60°, and 90°) were simulated to examine the effect of various conditions of multifidus on the lumbar spine. The default posture of each model was in an upright standing posture, and no obvious trunk flexion could be observed. Therefore, the default posture was treated as neutral spinal position with 0° trunk flexion. The segmental angles of the thoracolumbar spine in the sagittal plane were input into the models to calculate muscle activation and intervertebral joint reaction force. In summary, five conditions of multifidus and four flexional angles were considered (Figure 1), and all simulations were carried out on each of the 250 trunk musculoskeletal models. Therefore, 5000 (5*4*250) simulations were performed in the current study. 

OpenSim (Version 4.2) [35] was used for simulation, and Python scripts were coded for automatic adjustment of parameters, calculation of muscle activation, and joint reaction force. Since the contact module in OpenSim is not well suitable for facet joint simulation and most musculoskeletal models did not consider the contact force produced by the facet joints, only the flexion movement was considered, and the models were constrained in the sagittal plane. Static optimization in the OpenSim module was used for calculating muscle activation and muscle force [36]. Static optimization takes the known motion of the model as input and solves the equations of motion for the unknown joint torques subject to the following muscle activation-to-force conditions.

(1) $$\tau_{j}=\overset{n}{\underset{m=1}{\sum }}(a_{m}F_{m}^{0})r_{m,\;\;j}\;$$

where *n* refers to the number of muscles in the model, $a_{m}$ refers to the activation level of muscle *m*, and $F_{m}^{0}$ refers to its maximum isometric force. This method would compute the muscle activation to maintain the equilibrium state of trunk while minimizing the sum of squared muscle activation. Then, the joint reaction forces (compressive force and shear force) of the L5/S1 and L4/L5 levels were calculated and were represented in the local frame of the corresponding lumbar levels.

The load on the L4/L5 level was always used for model validation since several in vivo studies directly measured the IDP of this level [37,38]. To identify the influence of multifidus in lumbar load estimation, the compressive forces on L4/L5 level in various postures were compared with the results derived from in silico and in vivo studies [21,38]. Wilke et al. [38] measured L4/L5 IDPs from one subject in a set of daily activities including neutral position, 30° and 60° trunk flexion. They also measured the IDP in performing fingertip-floor movement, which was treated as 90° trunk flexion in the current study. To convert these values to compressive forces, the IDPs were multiplied by intervertebral CSA (1800 mm^2^) and then multiplied with a correction factor of 0.66 [21]. Fasser et al. [21] built a set of trunk musculoskeletal models and calculated L4/L5 compressive forces in upright standing and 30° flexion, which were also included for comparison. Due to the fact that some models after modification might not be able to calculate the outcomes, the success rate of simulation in each group was collected. Since the UBW is a subject-specific parameter, normalization can further reflect the relative force produced by each model. Therefore, the computed forces on the L5/S1 and L4/L5 levels were normalized by the UBW (Measured in Newtons) of each model for comparison. In each trunk posture, the normalized values from group 2–5 were compared with the control group (Group 1), respectively, using Wilcoxon matched-pairs signed-ranks test due to some non-normally distributed data. 

## 3. Results

**Figure 2:**
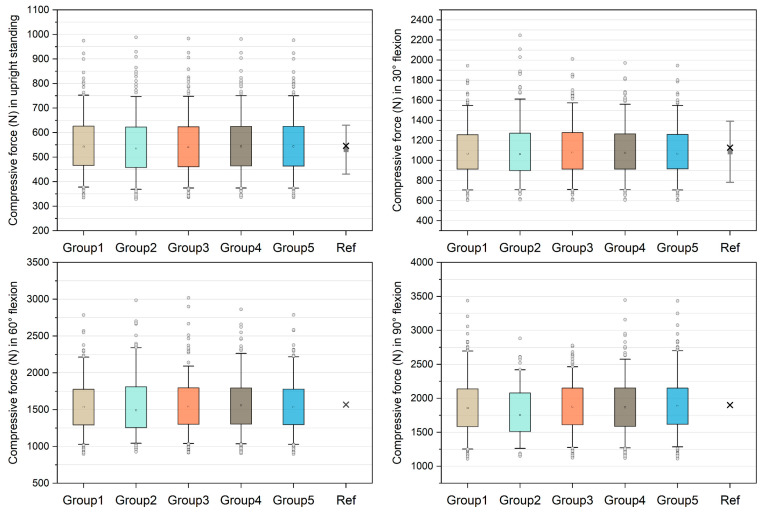
Unnormalized compressive forces on L4/L5 level compared with in silico and in vivo data [21,38]. The boxes represent the values between 25–75th percentiles, whereas the whiskers represent the values between 5–95th percentiles. In the reference (Ref) group, the grey squares with bars represent the means and standard deviations reported by [21], and the crosses represent the values derived from [38].

Figure 2 shows the comparison of unnormalized compressive forces on the L4/L5 level and the data derived from references [21,38]. The medians in group 1–5 models were 542.70 N, 535.63 N, 540.73 N, 543.58 N, and 543.33 N in upright standing and 1068.80 N, 1063.21 N, 1081.21 N, 1078.37 N, and 1069.03 N in 30° flexion, which approximate the in silico and in vivo data [21,38]. In 60° flexion, the medians were 1536.15 N, 1488.57 N, 1539.83 N, 1554.68 N, and 1536.34 N in group 1–5 models respectively. The median compressive forces in 90° flexion increased to 1856.00 N, 1753.41 N, 1877.42 N, 1866.54 N, and 1889.18 N in group 1–5 models, respectively. For each group of models, the success rates of simulation were reported in Table 1. In 90° flexion, the success rate of group 2 was 49.6%, followed by group 3 (84.4%), group 4 (89.6%), group 5 (92.8%), and group 1 (92.8%). 

The medians, 25th and 75th percentiles of the normalized compressive forces and shear forces on the L5/S1 and L4/L5 levels under four trunk flexional angles in the five groups were reported in Table 2. The statistical analyses show that the normalized compressive forces and shear forces on the L5/S1 and L4/L5 levels from group 2–5 were significantly different (*p* < 0.001) compared with group 1 in each posture.

**Figure 3:**
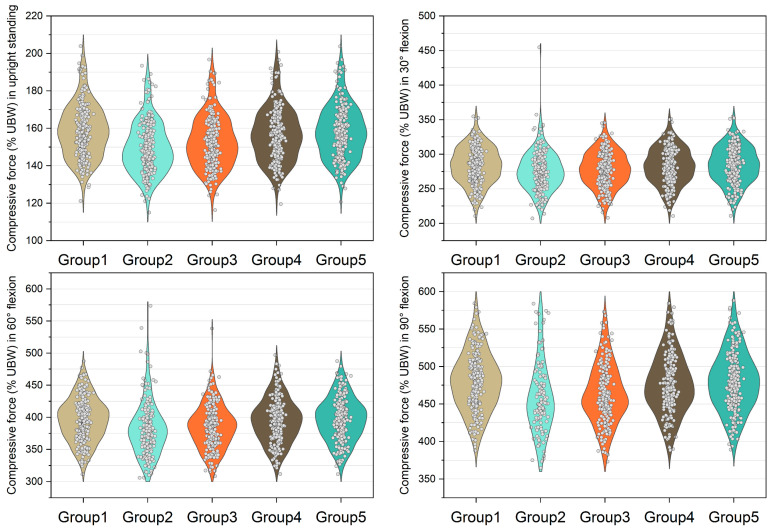
Normalized compressive force expressed by % upper body weight (%UBW) on the L5/S1 level. The grey circle denotes the value computed by each sample in each group.

**Figure 4:**
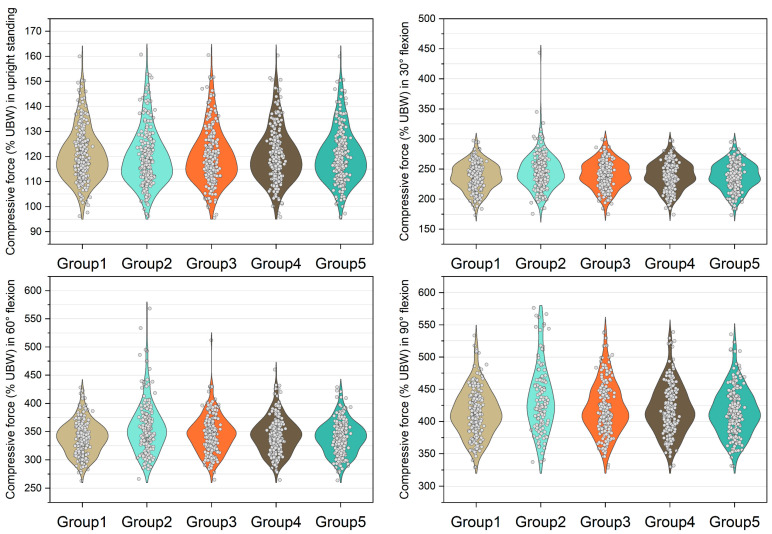
Normalized compressive force expressed by % upper body weight (%UBW) on the L4/L5 level. The grey circle denotes the value computed by each sample in each group.

**Figure 5:**
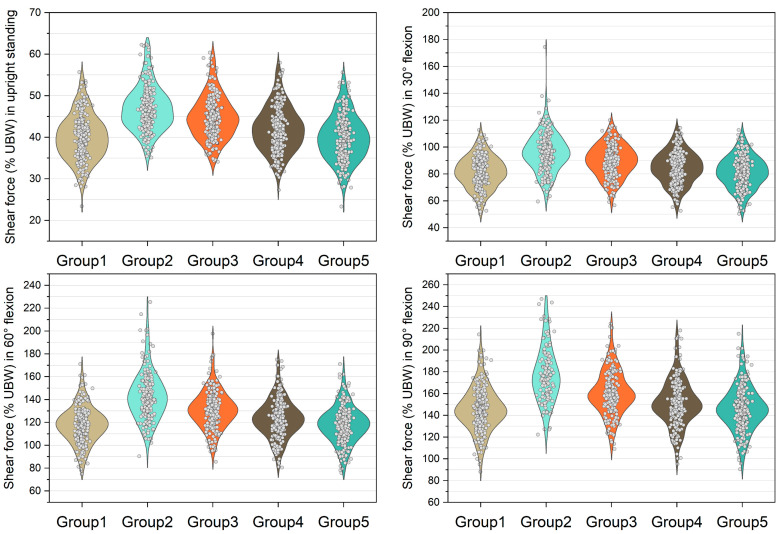
Normalized shear force expressed by % upper body weight (%UBW) on the L5/S1 level. The grey circle denotes the value computed by each sample in each group.

**Figure 6:**
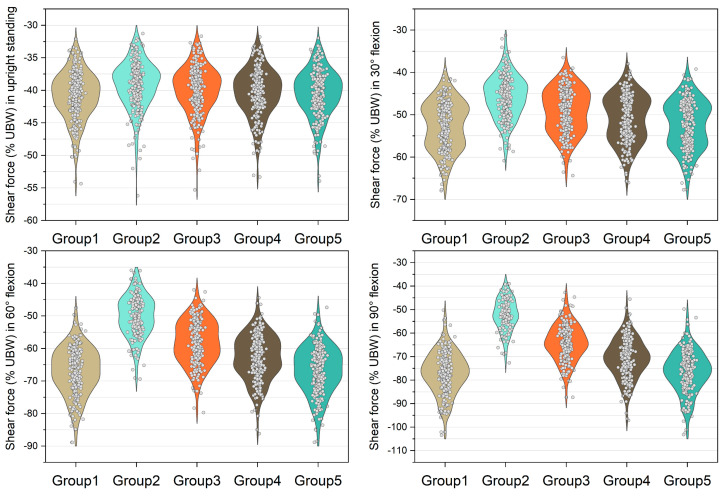
Normalized shear force expressed by % upper body weight (%UBW) on the L4/L5 level. The grey circle denotes the value computed by each sample in each group.

Figure 3, Figure 4, Figure 5 and Figure 6 show the normalized compressive and shearing forces on the L5/S1 and L4/L5 level under various conditions of multifidus and trunk flexional angles. In the models without multifidus modeling (Group 2), the normalized compressive forces on the L5/S1 level were significantly lower than the models with intact multifidus (Group 1) (*p* < 0.001) (Table 2, Figure 3). In extreme cases produced by group 2 models, the normalized compressive forces on the L5/S1 level were 455% UBW (30° flexion) and 574% UBW (60° flexion), which were 1.52 times and 1.36 times as large as the values computed by the corresponding models in group 1. In terms of the normalized compressive forces on L4/L5 level (Figure 4), group 2 models computed higher values (*p* < 0.001) than group 1 models in 30°, 60°, and 90° flexion postures. In extreme cases produced by group 2 models, the normalized compressive forces on L4/L5 level were 444% UBW (30° flexion), 568% UBW (60° flexion), and 576% UBW (90° flexion), which were 1.82 times, 1.63 times, and 1.13 times as large as the values computed by the corresponding models in group 1.

The medians of normalized shear forces on the L5/S1 level were the highest in the group 2 models, followed by group 3 and group 4 models under four postures (Figure 5). In extreme cases produced by group 2 models, the normalized shear forces on L5/S1 level were 174% UBW (30° flexion), 226% UBW (60° flexion), and 247% UBW (90° flexion). The absolute differences of the medians between group 1 and group 2 were 14.6% UBW (30° flexion), 23.3% UBW (60° flexion), and 28.5% UBW (90° flexion).

## 4. Discussion

In this study, the role of multifidus in the modeling of lumbar spine was investigated, and the influence of asymmetric multifidus atrophy of the lower back on the biomechanics of lumbar spine was explored. A set of musculoskeletal models were modified to achieve the objectives of this current study. The results demonstrated that incorporation of multifidus in the musculoskeletal models significantly influenced the load calculation of lumbar spine. The findings of this study may provide insights in the future modeling of the lumbar spine and highlight the role of multifidus in the biomechanics of the lower back.

The first objective of this study was to examine the function of multifidus in the modeling of the lumbar spine. Compared with in silico and in vivo studies [21,38], the unnormalized data shows that all five groups of models can predict approximate L4/L5 compressive forces whether the models included the multifidus or not. In the upright standing simulation, the 5th and 95th percentiles computed by group 1–5 were approximately 370 N and 750 N. This range is close to the results reported by Fasser et al. [21], in which the 95% confidence interval was between approximately 330 N and 730 N. In 30° trunk flexion, the 5th and 95th percentiles computed by group 1–5 were approximately 700 N and 1600 N, whereas a slightly larger range was reported by Fasser et al. [21] with 95% confidence interval between approximately 480 N and 1690 N. However, in terms of the medians and means, both of the results in the current study and Fasser et al. [21] were close to the values reported by Wilke et al. [38]. Therefore, for population-based trunk musculoskeletal models, multifidus may be neglected when building models to estimate average compressive forces on the lower lumbar levels in trunk flexion tasks. However, the success rate demonstrates that the multifidus is necessary for the software to find an optimal solution. Even when the multifidus was in half of its original maximum isometric force, the success rate was decreased by 4.4 % and 8.4% in 60° and 90° trunk flexion tasks, respectively. These results indicate that the multifidus should be incorporated in subject-specific models with small sample size, since neglection of this muscle may cause unsuccessful simulation. 

In terms of the normalized data, this study found that the incorporation of multifidus in the trunk musculoskeletal models had a minor effect on the compressive force on the lower lumbar levels in the simulation of upright posture. This can be explained by the fact that only a small amount force is needed to balance UBW in the upright position [39]. Therefore, the trunk model without multifidus can function well in equilibrating the load on the spine. This result also agrees with the previous studies investigating the morphology of the multifidus, in which the role of lumbar multifidus in the neutral zone was considered as a stabilizer of the spinal column [4,40,41]. Since the current study only considered static trunk postures with no perturbation, the multifidus had less effect in the neutral spine posture.

In contrast to the models with entire multifidus, the models without multifidus increased the variation in the compressive load estimation in the trunk flexion position (Figure 3 and Figure 4). In some cases, the models without multifidus may overestimate the compressive forces on the lower lumbar spine, the values of which were approximately twice the mean values (Figure 3). Similar to the results of compressive force, neglecting multifidus increased the variation in the estimation of shear force. In addition, the mean shear forces computed by the group 2 models were significantly different to that in the group 1 models (with entire multifidus) in all trunk flexion postures. These findings highlight the specific role of multifidus in the modeling of the lumbar spine. According to the muscle attachment sites [3], the deepest multifidus fascicles only connect two adjacent vertebrae in the dorsal side of spinal column, while other multifidus fascicles run from one vertebra to the second or several levels of vertebra above [42]. Due to these anatomical features, multifidus is able to provide an extension moment with redundancy that helps to fine-tune the segmental balance of the spine. This important function cannot be totally replaced by the other erector spinae muscles including longissimus and iliocostalis. Therefore, the results of the current study support the view that it is necessary to incorporate the multifidus in the modeling of trunk musculoskeletal model for better estimation of lumbar loading.

The second objective of this study was to investigate the influence of asymmetric multifidus atrophy on the biomechanics of lumbar spine. This study found that asymmetric multifidus atrophy had minor effect on the lower lumbar spine in terms of the compressive force and shear force. This result indicates that asymmetric multifidus atrophy can be compensated by other back muscles, and thus, it may have negligible effect on the lower lumbar spine in the flexion posture. This result may partially explain the reason why no correlation was found between lumbar multifidus thickness and disability in chronic low back pain patients [32]. In 90° trunk flexion, this study found that the group five models (asymmetric multifidus atrophy on L4/L5 level) had slightly higher success rate of simulation (92.8%) compared with the group 4 models (89.6%). This result suggests that the multifidus across L4/L5 level may be more important than the multifidus across L5/S1 level in maintaining balance of lower lumbar spine in 90° trunk flexion posture. 

This study also had limitations. Firstly, only the trunk postures in the sagittal plane were considered in the current study. This was due to the reason that the existing trunk musculoskeletal models were not well suitable in simulating movement involving facet joint contact, e.g., extension, axial rotation. Future musculoskeletal models may incorporate the facet joint to improve the fidelity of simulation, which can be used to simulate more scenarios of trunk movements. Secondly, this study only simulated the static trunk postures. Future studies will be encouraged to simulate more dynamic trunk movements when the kinematics of each lumbar vertebrae is available.

Except for the above-motioned limitations, this study identified the specific mechanical role of lumbar multifidus in fine-tuning the lumbar loads. Since this study used a set of biomechanical models for analyses, several factors need to be taken into consideration when interpreting the results. Firstly, this study included 250 subject-specific trunk musculoskeletal models, and each model was modified into five models in terms of various multifidus configurations. Therefore, the mechanical results are robust due to the sample size. On the other hand, this relatively large number of models may affect the results of the study. Although significant differences can be found when comparing the values between group 2–5 and group 1, some of these may have less significance in practice due to the low absolute differences between groups. Therefore, violin plots were applied in the current study to reflect the data distribution and the extreme cases, and to help draw the main conclusion of this study. Finally, this study was based on biomechanical simulation. Future studies may combine subject-specific modeling with in vivo measures of muscle activation to further confirm the role of multifidus in low back pain patients.

## 5. Conclusions

This study found that incorporating the multifidus in the trunk musculoskeletal modeling had an impact on the compressive force and shear force estimation of lower lumbar spine. It is important to model multifidus to increase the success rate of simulation and decrease the incidence of overestimation of compressive load on the lumbar spine. Asymmetric multifidus atrophy had a minor effect on the lower lumbar spine in the sagittal plane. The results highlighted the fine-tuning ability of multifidus in equilibrating the loads on the lower back and the necessity of incorporating multifidus in trunk musculoskeletal modeling.

## Figures and Tables

**Table 1:** Success rate (Number of successful simulations/number of simulations performed in each group) in various trunk flexion tasks. Each group contains 250 models, and thus, 250 simulations were performed in each group. When the static optimization could find an optimal solution, it was treated as an unsuccessful simulation.

Flexional Angle	Group 1	Group 2	Group 3	Group 4	Group 5
Upright	100% (250/250)	99.2% (248/250)	100% (250/250)	100% (250/250)	100% (250/250)
30°	100% (250/250)	91.6% (229/250)	100% (250/250)	100% (250/250)	100% (250/250)
60°	100% (250/250)	76% (190/250)	95.6% (239/250)	99.6% (249/250)	100% (250/250)
90°	92.8% (232/250)	49.6% (124/250)	84.4% (211/250)	89.6% (224/250)	92.8% (232/250)

**Table 2:** Normalized compressive force and shear force expressed by % upper body weight (UBW) with medians, 25th, and 75% percentiles on the L5/S1 and L4/L5 levels. Values from group 2–5 were compared with group 1 using Wilcoxon matched-pairs signed-ranks test (* indicates *p* < 0.001).

Force	Level	Flexional Angle	Group 1	Group 2	Group 3	Group 4	Group 5
Compressive force	L5/S1	Upright	157.9 (147.6, 168.2)	147.9 * (139.3, 158.8)	151.3 * (142.5, 162.5)	156.0 * (145.4, 166.0)	156.8 * (147.0, 167.4)
		30°	285.3 (265.7, 304.5)	273.7 * (257.0, 293.6)	278.9 * (260.0, 297.9)	283.5 * (264.3, 303.2)	285.2 * (265.7, 304.5)
		60°	396.0 (371.1, 418.4)	378.7 * (354.5, 400.4)	386.5 * (360.7, 406.4)	394.9 * (370.4, 417.2)	396.3 * (372.4, 419.3)
		90°	479.5 (452.4, 506.3)	450.7 * (427.3, 483.8)	458.9 *( 439.5, 488.0)	472.2 * (449.8, 502.0)	478.9 * (453.5, 507.0)
	L4/L5	Upright	118.9 (113.0, 126.0)	117.9 * (111.8, 125.3)	118.3 * (112.3, 126.0)	118.7 * (112.7, 126.0)	118.5 * (112.6, 125.8)
		30°	238.2 (222.7, 2.54.0)	243.3 * (228.0, 2.61.6)	240.3 * (225.7, 257.7)	239.4 * (224.9, 256.1)	238.6 * (223.5, 254.3)
		60°	338.6 (318.3, 357.3)	351.9 * (326.6, 377.0)	345.2 * (322.4, 364.8)	343.0 * (320.9, 362.6)	339.0 * (318.9, 358.4)
		90°	411.5 (388.0, 436.6)	424.4 * (398.2, 463.1)	414.2 * (392.7, 447.7)	414.2 * (392.2, 443.3)	412.6 * (388.3, 439.0)
Shear force	L5/S1	Upright	39.6 (36.1, 43.7)	46.6 * (43.2, 50.0)	44.4 * (41.0, 47.9)	41.9 * (38.2, 45.4)	39.4 * (36.1, 43.4)
		30°	81.4 (73.8, 89.4)	96.0 * (87.7, 104.0)	90.0 * (82.2, 97.9)	84.9 * (77.0, 93.0)	81.5 * (73.9, 89.4)
		60°	118.0 (108.3, 127.2)	141.3 * (129.3, 154.3)	130.8 * (121.2, 140.5)	122.7 * (113.6, 133.2)	118.5 * (108.9, 127.6)
		90°	145.0 (134.0, 158.1)	173.5 * (159.9, 191.7)	159.2 * (149.8, 174.0)	150.0 * (140.9, 164.4)	145.5 * (134.0, 157.9)
	L4/L5	Upright	−40.5 (−43.0, −38.3)	−38.6 * (−41.1, −36.8)	−39.4 * (−41.8, −37.5)	−40.0 * (−42.3, −37.9)	−40.1 * (−42.5, −37.9)
		30°	−52.3 (−57.1, −47.9)	−45.3 * (−49.2, −42.3)	−48.8 * (−53.2, −44.8)	−50.3 * (−55.2, −46.2)	−52.1 * (−56.9, −47.7)
		60°	−66.9 (−71.7, −61.6)	−49.8 * (−54.1, −45.6)	−57.9 * (−63.0, −53.1)	−62.7 * (−67.4, −57.8)	−66.4 * (−71.2, −61.4)
		90°	−76.3 (−82.3, −71.2)	−52.3 * (−56.4, −46.9)	−64.4 * (−69.1, −59.1)	−70.6 * (−75.5, −65.6)	−75.6 * (−81.5, −70.9)

## Data Availability

All data from this study are available from the authors upon reasonable request.
